# Patient Endorsement of the Outcome Measures in Rheumatology (OMERACT) Total Joint Replacement (TJR) clinical trial draft core domain set

**DOI:** 10.1186/s12891-017-1464-x

**Published:** 2017-03-15

**Authors:** Jasvinder A. Singh, Michelle Dowsey, Peter F. Choong

**Affiliations:** 1 University of Alabama at Birmingham, 510 20th Street S, Faculty Office Tower 805B, Birmingham, AL 35294 USA; 2 Birmingham Veterans Affairs Medical Center, 700 19th St S, Birmingham, AL 35233 USA; 3 Department of Surgery, University of Melbourne, St. Vincent’s Hospital, Melbourne, Australia

## Abstract

**Background:**

A patient- and surgeon-Delphi-derived Outcome Measures in Rheumatology (OMERACT) draft core domain set for total joint arthroplasty (TJR) trials was recently developed. Our objective was to obtain further patient stakeholder endorsement of draft core domain set for TJR clinical trials.

**Methods:**

We surveyed two patient groups: (1) OMERACT patient partners; and (2) patients who had undergone hip or knee TJR. Patients received an introductory email with explanations about the core domain set and instructions to rate the core domains, i.e., important aspects, of OMERACT TJR clinical trial draft core domain set. Rating was on a nominal scale, where 1–3 indicated a domain of limited importance, 4–6 an important, but not critical domain, and 7–9 a critical domain. We used Mann–Whitney test (a non-parametric test) to compare the distribution of ratings between the two groups.

**Results:**

Thirty one survey participants from the OMERACT patient partner group and 118 knee/hip TJR patients responded with response rates of 66 and 80%, respectively. Majority of the survey respondents were female, 87 vs. 53%, and were 55 years or older, 57 vs. 94%. Median (interquartile range [IQR]) scores for six core domains by OMERACT and knee/hip TJR patient groups were, respectively: pain, 8 [8, 9] and 9 [8, 9]; function, 9 [8, 9] and 9 [8, 9]; patient satisfaction, 8 [8, 9] and 8 [7, 9]; revision surgery, 7 [7, 8] and 7 [5, 9]; adverse events, 8 [7, 9] and 8 [6, 9]; and death, 9 [6, 9] and 9 [4, 9]. No statistically significant differences in rating were noted for any of the six core domains between the two groups (*p* ≥ 0.31). Among the additional domains, ratings for patient participation did not differ by group (*p* = 0.98), but ratings for cost were significantly different (*p* = 0.005). Patients provided qualitative feedback regarding core domains, and did not propose any modifications to the draft core domain set.

**Conclusions:**

Two separate patient stakeholder groups endorsed the OMERACT TJR draft core domain set for TJR trials.

**Electronic supplementary material:**

The online version of this article (doi:10.1186/s12891-017-1464-x) contains supplementary material, which is available to authorized users.

## Background

Total Joint Replacement (TJR) is a very successful and cost effective surgical treatment for refractory end-stage arthritis in patients who report pain and/or functional limitation. Majority of the patients report impressive improvements in pain and function with this surgery [1, 2], while a small proportion have an unsatisfactory outcome from TJR [3, 4], including persistent pain and functional limitation [5–10]. Although hip and knee TJR are the most common joints replaced currently, we realize that TJR for other joints is also becoming more common in the recent years [11–15]. While the rapid advances in implant design and surgical techniques have occurred for TJR, TJR trial reporting has not kept pace with this quick evolution. Development of valid outcome measures, universal adoption and harmonization continues to be a big challenge. As a result, patients and surgeons have not been able to effectively capitalize on the large and growing body of data for cross study comparisons. Moreover, the heterogeneity in outcome measures used in TJR trials [16] prevents meta-analysis and literature synthesis and this is compounded by the limited validity of several of the outcome instruments being used [17]. Recent studies confirmed that these challenges continue in knee TJR trials [18, 19]. In 2006, our group recognized the need for consensus and harmonization of reporting of TJR trials [20]. We have built capacity and capability by undertaking several systematic reviews and building collaborations, which have continued over the last decade. This has led to the formation of an international Working group (WG) on TJR clinical trial outcomes that is now an orthopedist-patient-rheumatologist-methodologist multi-stakeholder collaborative [21].

Using the Outcomes Measures in Rheumatology (OMERACT) filter for developing core measurement sets, we developed a multi-stakeholder endorsed TJR trial draft core domain set, based on systematic literature reviews, followed by input from patients and surgeons using a Delphi process [21] and mailed surveys. The OMERACT TJR draft core domain set (for any joint) included the following: Pain, function, patient satisfaction, revision, adverse events, and death [21]. The OMERACT TJR trial draft core domain set was developed based on systematic literature reviews, several multi-stakeholder discussions at OMERACT and various orthopedic meetings that included leaders in arthroplasty registries, trials and outcome science, Delphi surveys and a face-to-face discussion of each potential core domains with patients, orthopedic surgeons, methodologist, physical and occupational therapists and rheumatologists, details provided elsewhere [21]. This outcome draft core domain set is consistent with previous systematic reviews of patient outcomes after TJR and qualitative studies of patient perspective of TJR outcomes [3, 16, 22–24]. In a subsequent consensus-building exercise, we achieved a complete consensus for this draft TJR core domain set with two independent groups of orthopaedic surgeons, who endorsed all six core domains [25].

As part of our long-term objective to develop and implement a TJR core measurement set, the aim of the current study was to obtain further endorsement of the OMERACT TJR trial draft core domain set, for any TJR for any joint, as defined previously [21]. We aimed to survey new patient groups, who are representative internationally, and are independent of the patient group that helped us develop the original draft core domain set. Another objective was to identify whether these independent patient surveys find evidence for modifying our preliminary OMERACT TJR trial core. This study describes the process of obtaining further endorsement of the OMERACT TJR trial draft core domain set for any joint from a wider patient audience.

## Methods

We surveyed two groups of patients. The first group included the OMERACT patient research partner (PRPs), a group of patients with rheumatic diseases (predominantly arthritis, but also including patients with immune conditions) who had attended one of the biannual OMERACT meetings including the 2016 OMERACT meeting at Whistler, Canada (*n* = 47). The second group was a cohort of patients who had undergone hip or knee replacement at St. Vincent’s Private Hospital (SVPH), Melbourne, Australia between January 2013 and December 2014 (*n* = 147). This cohort was identified through the St. Vincent’s Melbourne Arthroplasty Registry (SMART), approved by the Human ethics committee at St. Vincent’s Hospital, Australia (HREC-A 100/14; there is no published protocol). We recruited patients after knee or hip TJR, since our goal was to develop a core domain set for TJR trials, and therefore recruiting pre-operative patients does not address our main question.

We developed and pre-tested a survey questionnaire, which was reviewed by several colleagues with previous TJR attending the OMERACT meeting, study team members as well as patients in the SMART registry. Subsequently, it was iteratively modified, based on the feedback, including clarification of the questions and simplification of the cover letter, the background and the purpose of the questionnaire. The survey queried patient characteristics and the importance of each domain of the OMERACT TJR trial draft core domain set, including the six core areas/domains (pain, function [ability to function in society, work; work productivity, employability; disability; work disability], patient satisfaction [satisfaction with the outcome, satisfaction with the procedure], revision surgery, adverse event and death) and two optional areas/domains (patient participation in life/social activities, cost).

Patients rated each core domain on a 1–9 scale, developed by the GRADE group, used by us for rating outcomes in previous studies of development of treatment guideline [26, 27] and our previous studies ranking disease core domains [25, 28]: 1–3 indicated a domain of limited importance, 4–6 an important, but not critical domain, and 7–9 a critically important domain. Surveys were emailed to both groups of patients. In addition, a hard copy was mailed to patients from the SVPH cohort without email addresses (*n* = 23). Patients completed these annonymized surveys using Survey Monkey link sent to their emails or a mailed copy. Those not responding received two reminders at least 1-week apart.

We defined the purpose of patient surveys in our previous paper, which was to assess whether patients could achieve consensus on the current core domain set for TJR trials, seek patient endorsement of the core domain set and in case of any suggestions for modification/s, the reasons for change in core domains [21]. We defined the consensus as follows. If both groups of patients would rate each core domain between 7–9, i.e., critical, this would constitute complete consensus. If both patient groups rated a majority of the six core domains, but not all, as critical, we would consider this as incomplete consensus. In case of incomplete consensus, we would have further discussion with patients and we would plan to modify the draft TJR trial core domain set for any joint. If the patient groups achieved complete consensus on core domains, given the previous complete consensus by orthopaedic surgeons [25], the draft TJR core domain set would be considered endorsed by both patients and orthopaedic surgeons at this stage. We calculated summary statistics including proportions and medians (interquartile range), as applicable, separately for the OMERACT PRP and the SVPH patient cohorts. We used a non-parametric test, Mann–Whitney to compare the distribution of ratings between the two groups and considered a p-value <0.05 to be statistically significant.

## Results

Survey response rates were good to excellent, 66% for OMERACT PRPs and 80%, for SVPH patients. The two cohorts differed in gender, age and diagnosis (Table 1). Compared to the SVPH cohort, OMERACT PRP group had greater proportions of females, 87 vs. 53%, those with a diagnosis of RA, 4 vs. 54%, and was younger (Table 1). 85% of OMERACT and 97% of SVPH patients had a diagnosis of arthritis. A key difference was that 27% of OMERACT and 100% of the SVPH cohort had had a joint replacement.

**Table 1:** Participant characteristics of the two patient cohorts

	OMERACT Patient research partner (PRP) Cohort survey (*N* = 47)	Total Joint Replacement SVPH Cohort (*N* = 147)
# Survey participants	*N* = 31 (66%)	*N* = 118^a^ (80%)
% Male^b^	4 (13%)	50 (47%)
Age category^b^		
18-24	0	0
25-34	1 (3%)	0
35-44	3 (10%)	1 (1%)
45-54	9 (30%)	5 (5%)
55-64	11 (37%)	26 (25%)
65-74	6 (20%)	36 (34%)
≥75	0	37 (35%)
Missing	2 (7%)	2
Had a joint replacement	13 (42%)	107 (100%)
Type of arthritis		
Osteoarthritis	2 (7%)	93 (87%)
Rheumatoid arthritis	15 (54%)	4 (4%)
Other inflammatory arthritis	7 (26%)	7 (6%)
Joint aches/pains, no arthritis diagnosed or no joint aches/pains	4 (15%)	3 (3%)

^a^Of the 147 patients who received the survey (email, *n* = 124; mailed survey, *n* = 23), 118 responded (email, *n* = 109; mailed survey, *n* = 9), of which 107 were usable (email, *n* = 99; mailed survey, *n* = 9). Therefore, most proportions are out of 107 (73% valid surveys)

^b^Missing responses for variables for OMERACT PRPs vs. TJR SVPH cohorts: sex: 0 vs. 2; age, 0 vs. 2

Despite these differences, both cohorts rated OMERACT TJR clinical trials core domains similarly (Table 2). On a 1 to 9 scale, all six-core domains were rated as critical domains (score 7–9); the additional domains of cost and participation were scored important to critical (scores 6–8) (Table 2). None of the core domain set ratings differed significantly between the two groups (Table 2). For additional domains, participation ratings were not statistically significantly different by group but rating for cost as an additional domain was statistically significantly higher for the SVPH Cohort compared to the OMERACT patient cohort (*p* = 0.005; Table 2).

**Table 2:** Patient Consensus on OMERACT TJR Clinical Trial Core Areas/Domains

	OMERACT PRP (*n* = 31) Median [IQR]	SVPH Cohort (*n* = 118^a^) Median [IQR]	*p*-value (Mann–Whitney test)
**Core Domains to be reported in every TJR clinical trial**			
Joint Pain	8 [8, 9]	9 [8, 9]	0.75
Function or functional ability (ability to function in society, work; work productivity, employability; disability; work disability)	9 [8, 9]	9 [8, 9]	0.31
Patient Satisfaction (satisfaction with the outcome, satisfaction with the procedure)	8 [8, 9]	8 [7, 9]	0.86
Revision surgery (including reoperation)	7 [7, 8]	7 [5, 9]	0.97
Adverse events (total and specific)	8 [7, 9]	8 [6, 9]	0.84
Death	9 [6, 9]	9 [4, 9]	0.70
**Additional domains for consideration for reporting in TJR clinical trials**			
Patient Participation	8 [6.5, 8]	7 [6, 9]	0.98
Cost	6 [5, 7]	7 [6, 8]	0.005

^a^118 responded, of which 107 had valid, analyzable responses

We received additional free text comments, which provided insight into defining these core domains further and suggestions as to which instruments we should consider for the next step in the development of the TJR clinical trial core measurement set (Additional file 1). Additional areas brought up for consideration included several that are either included as core domain or are closely related to it: [1] life of an implant (same as the core domain, revision); [2] Recovery and rehabilitation (similar to pain, function or functional ability, satisfaction); [3] Independence (similar to function or functional ability).

## Discussion

This study describes patient consensus and endorsement of the OMERACT TJR trial draft core domain set, which was first developed and endorsed with multi-stakeholder input in 2014, including patients and surgeons, after completing several systematic reviews that helped identify potential core domain set [21]. As described in our previous study, the current study completes step 5, i.e., patient endorsement of the preliminary OMERACT TJR trial core domain set, such that we now have an OMERACT TJR trial draft core domain set. We specified a priori that patients and surgeons were our two key stakeholders [21]. Therefore, the completion of this patient survey that followed surgeon survey [25], which was published in the interim, provided us with consensus we were aiming for TJR trial core domain set. Both surgeons and patients have achieved consensus on TJR trial core domain set consisting of six core domains, and no modification is needed, based on the results of these two studies. Several findings in this study merit further discussion.

A key aspect of core domain development is its endorsement by a wider group of stakeholders. The OMERACT process uses cutting-edge methodological science and rigor and is a data-driven and consensus-based development of outcome instruments for clinical trials. The OMERACT filter 2.0 provides a well-described framework for the development of core domain and core measurement sets [29]. Although this process of developing a multi-stakeholder group and developing the core domain set has taken a long time since we started a decade ago, we have followed the OMERACT methodology rigorously [21]. We believe that this rigor will ultimately help with a wider acceptance and uptake of the TJR trial core domain set. We have now developed core domain set for TJR trials based on the OMERACT filter 2.0 [29, 30].

Our current study shows that two groups of patients endorsed the OMERACT TJR trial preliminary core domain set, rating all six domains as critical. Despite differences in patient characteristics, the ratings by two cohorts were similar, which is a testament to a rigorous process that we have used to develop this draft core domain set. This draft TJR trial core domain set has now been endorsed by several independent groups of patients and orthopaedic surgeons, two key stakeholders.

The methodology for core domain set development for TJR trials was a standard method of defining every domain that is core to the condition, i.e., there is no prioritization [29–31]. Therefore, the patient survey elicited responses whether each of the core/additional domains was core to TJR outcome or not at its own merit, without asking them to prioritize the domains. The last two steps in this process are dissemination and a wider endorsement of the core domain set by orthopedic surgeons, and the development, validation and endorsement of a TJR trial core measurement set by a multi-stakeholder group, and possible co-branding of this OMERACT TJR trial draft core domain set and core measurement set by interested orthopedic societies.

This study shows that a few patients identified additional potential domains, including recovery and rehabilitation. It may be debated whether these are already represented by the core domains currently included i.e. pain, function or functional ability and patient satisfaction. The definitions for function [ability to function in society, work; work productivity, employability; disability; work disability] and satisfaction [[satisfaction with the outcome, satisfaction with the procedure] and domain of pain indicated to us that recovery and rehabilitation are processes that are intertwined with these core domains and perhaps already captured to some extent with these domains. However, in order to ensure appropriate rigor and inclusiveness, further discussions are required with wide stakeholder input, regarding whether these should be optional domains or be on research agenda.

We noted that real-world concerns were also brought up by a few participants related to TJR that are not relevant for TJR clinical trial reporting. Examples include access to and wait times for TJR, which are related to health care access and are country and setting-specific contextual factors, likely relevant for observational studies and/or public policy discussions. These are important policy concerns, but not relevant to TJR clinical trial core domain selection.

Our study has several limitations. Survey response rates were 66 and 80%, but we do not have non-responder characteristics to understand how non-response influenced our findings. However, the high response rate should give readers more confidence in these findings. To strengthen our conclusions, we attempted to get a wide perspective by polling representative groups, including patients who had undergone knee/hip TJR. We surveyed only adults and focused on knee or hip TJR trials. These findings cannot be generalized to TJR that involve other joints. Study strengths included the assessment of the validity of core domain sets in two patient populations, an international representativeness of sample, robustness of findings despite patient characteristic differences in survey participants, and a high response rate.

## Conclusions

In conclusion, two patient groups in separate surveys have now endorsed the OMERACT TJR trial draft core domain set. The OMERACT TJR draft core domain set was previously developed with multi-stakeholder input, including patients and surgeons. All current core and additional domains were endorsed as they are, without any modification. With a parallel consensus from orthopaedic surgeons showing consensus for the 6 domains (In a different study of the same core domains), both patients have achieved consensus on TJR trial draft core domain set consisting of six core domains, and based on the results of these two studies, no modification is needed. We also obtained important feedback regarding these domains, which will be instrumental in our next steps of instrument selection for each of the core domains. The planning is currently underway to seek a wider dissemination to and/or endorsement by general membership of arthroplasty surgeon societies and/or OMERACT. If the draft core domain set is also endorsed by a wider orthopedic society membership, we will proceed with the development of first consensus-based data-driven TJR trial core measurement set.

## Additional file

Additional file 1: Additional Comments from Delphi Process. This file provides the free text comments from both the OMERACT group as well as the St. Vincent's Hospital Cohort regarding the core domains and other suggested domains. (DOCX 127 kb)

## Abbreviations

OMERACT: Outcome Measures in Rheumatology
PRPs: Patient research partner
SMART: St. Vincent’s Melbourne Arthroplasty Registry
SVPH: St. Vincent’s Private Hospital
TJR: Total Joint Replacement
WG: Working Group
