# Supplementary material for: Patient Endorsement of the Outcome Measures in Rheumatology (OMERACT) Total Joint Replacement (TJR) clinical trial draft core domain set
Source: BMC Musculoskelet Disord. 2017 Mar 15;18:111. doi: 10.1186/s12891-017-1464-x (PMC5353795; doi:10.1186/s12891-017-1464-x)
Supplement: Additional file 1: — Additional Comments from Delphi Process. This file provides the free text comments from both the OMERACT group as well as the St. Vincent's Hospital Cohort regarding the core domains and other suggested domains. (DOCX 127 kb) [file 12891_2017_1464_MOESM1_ESM.docx]

**Supplementary Files**

**Supplementary File 1. Additional Comments from Delphi Process**

| **OMERACT Patient Research Partner group** | |
| --- | --- |
|  |  |
| Main Core Domains (rating on a 1-9 scale*) | Additional domains |
| Not sure of relevance of "death" above. This is not specific enough - death through complications? Through joint replacement surgery? This core domain needs to be more specific. | Not sure what 'cost' means here. Are we talking about the estimated cost of each specific type of surgery, or the actual costs in each case, even if infections or complications arise. I would like to see 'infections following surgery' reported in the core set too |
| All of the above are very important and it is hard to not rank them all a nine. One would hope revision surgery is not necessary.. | Surface appearance of the body after the procedure (rating, 7)--i.e. do both sides match, Effects on other joints- 9 i.e. the back is now a problem; |
| Recovery time needed- (ranking, 7); it could be part of patient satisfaction but hospitals would like this information too as a separate item. How invasive a procedure- (ranking, 9); a problem in how to measure this but a scale could be designed. | Re-gaining as much independence as possible improvement in function/mobility |
| Time scale from diagnosis to full recovery (rating, 6) | Access/availability of the intervention 7 |
| Range of movement / motion | How long the arthroplasty lasts, e.g. seven years, 15 years, beyond 20 years. |
| From my perspective death is not relevant. (hand OA or possibly knee OA) | Waiting time from the point when the patient requests medical intervention. (rating, 8) Recuperation time. (rating, 7) |
| Continued reliance on pain medication postop. (rating, 6) | I am in "shared decision making" SIG hence my high score for patient participation. |
|  |  |
| **Queens Hospital Cohort survey (n=107)** | |
| More consideration given too the pain relief provided. The adverse effects of blood clot injections. | cost - what is being asked here? Is it that cost may be relative to the outcome? Or is that cost is important to the patient? I have answered based on the former. Patient participation - what does this mean? We have assumed it means how involved was the patient in decision making prior to the surgery. |
| Ongoing scar pain due to staf infection and consequent flushing out of wound (twice) | Both of the above are important coming from someone who has continual hip pain (with extenuating circumstances) to those unable to fund such procedures. |
| I suppose that Adverse events may cover it, but I experienced months of insomnia, and needed sustained physiotherapy in order to manage pain and achieve mobility | Joint performance after surgery, patient usage of hip, changed to gait etc. ability to perform previous sporting activities |
| Quality of nursing: After my first operation this was extremely satisfactory. However, after my second hip replacement, twelve months later, I felt that not enough care was taken in inspecting my wounds. e.g. after the removal of my drainage tube ( much sooner than after my previous operation) the wound was not checked for at least twelve hours by which time it had leaked through my bedding. I had to alert the nurse to the fact that I felt wet/sweaty on my back. It continued to weep after I had been transferred to Re Hab. | The gap between charge and medical rebates very high and burdensome. However the results for me far outweigh the outlay |
| Recovery times | Post operative care - physio, water therapy. |
| Physical fitness before procedure. (rating, 8) | The approximate life of joint replacement . rate 7 |
| Links to non surgical adverse effects eg affect on memory of anaesthetic or blood loss. | Convenience -location/timing of surgery/recovery |
| I have had a Knee & Hip replacement recently and not sure which to comment on. My Knee has recovered well, slightly aches at times. Since overextending after my hip replacement, I suffer extreme pain from my glute to my foot and after extensive rehab and physio I still have the constant pain and find it debilitating to walk with the pain causing me to limp all the time. It feels like nerve and muscle pain. | I think patient participation is important in giving an overall understanding of the surgery and the implications for future health and lifestyle. I do not want lots of information about the details of the surgery, risks, as if you have chosen the Surgeon with care you have confidence that the operation will be performed with skill and care. |
| My left knee had had arthritis gradually worsening for about 15 years. At age 70, I had a horse accident, which resulted in a double hip fracture on the left side. Surgery at the Northern Hospital was excellent, but my arthritic knee meant I had to keep using a walking stick, at which stage I had a knee replacement. Both operations were brilliant and I have had no trouble and no pain from either. | In my work as a psychologist (now retired), I am aware that many people are unable to afford private hospital cover and wait for a long time, for public hospital treatment. I think that patient involvement is really important and could include education and support, if needed. |
| After surgery perhaps patients should be alerted to the possibility of complications such as bursitis. | Rehabilitation experience and duration |
| I am more than satisfied with the outcome of the two joint replacements however, I still find full mobility (I.e. Climbing stairs and high curbs etc.) difficult to manage but I will overcome this in time. I used to have a problem walking up hills, but now my legs are the same length and through constant exercise and strength training, it has made this easier to do. | I found going through both the hip and knee procedures and recovery very traumatic, but considering the path I was heading down in the long run, the outcome has been worth it. |
| Very important that patient and surgeon and anaesthetic staff treat every patient individually and listen to patient especially in regard to drug sensitivities and other concerns (rating, 9) | After surgery I had to go every day for a month for an anti-blood clotting injection. I later found out I could have had a tablet for this which would have been a lot more acceptable to me. |
| I had a very bad reaction to the drugs administered to me and went through a psychotic episode whereby I thought I was being hunted down and I broke out of the hospital and was gone for two hours approx. which caused great distress to me my family and hospital staff. As I am unused to taking any pain medication I think the drugs should have been monitored more carefully. I also bled badly in the operation possibly due to the fact that I am a cancer patient. However my operation was successful and although I have some joint pain (possibly because other hip needs doing) overall I am able to do most things and try to keep up a good exercise regime of swimming bike riding walking (a bit painful sometimes) etc. | My immediate response was that neither of these two issue were of critical importance but on reflection. I realise that they are critical. In the past many elderly patients didn't want to know what was happening they just wanted "ít" fixed but with changing generations this is no longer the case. Regarding cost, joint replacements make a huge difference to quality of life and need to be accessible to as many as possible. Therefore cost should be include in any clinical trial to ensure that cost and efficacy can be evaluated |
| Rehabilitation | It's a bit costly |
| Co-morbidities that may effect long term outcome of joint replacement would be helpful to the trials | I think that all avenues are considered before joint replacement , especially as you here and read of different options and trials available so that joint replacement is the only solution available when the patient goes to surgery |
| Function or functional ability could be split up into I. Degree of mobility ii. Independence in ADL iii. Ability to work productively. | Rehab of joint once able to use joint, I found critical to recovery! My rating is 9 |
| Our expectations re: surgery will always be high on the 1-9 but there are always reasons such as age,, health, willingness to do all physio and exercise required to get full use that need to be considered as well. | A mandatory exercise rehabilitation & ongoing exercise programs for the patient going home following joint replacements |
| The amount of time patients have had to wait for their surgery, and the effect of this wait-time on their quality of life, are very important factors. This is a strange survey - hard to work out where it's coming from. Intro needs rewriting - better explanation, less repetition. | Whether the underlying cause of the joint failure will result in the deterioration of other joints in the future. |
| I suggest that over eighty (assuming the replacement operation) that death is of less consideration | Clearly patient satisfaction and experience are important to assessing success of joint replacement, but I don't understand the methodology for including it. Hard to assess cost in an international survey where approaches to paying for healthcare vary so widely |
